# Novel Mutation With Literature Review WW Domain-Containing Oxidoreductase (WWOX) Gene

**DOI:** 10.7759/cureus.25003

**Published:** 2022-05-14

**Authors:** Ghassan Sukkar, Razan M Alzahrani, Bsaim A Altirkistani, Rayan S Al lohaibi

**Affiliations:** 1 Department of Pediatrics, Ministry of National Guard - Health Affairs, Jeddah, SAU; 2 College of Medicine, King Saud Bin Abdulaziz University for Health Sciences, Jeddah, SAU; 3 College of Medicine, Ibn Sina National College, Jeddah, SAU

**Keywords:** novel mutation, wes, whole-exome sequencing (wes), ww domain-containing oxidoreductase (wwox) gene, homozygous

## Abstract

Genetic alterations in the WW domain-containing oxidoreductase (*WWOX*) gene cause autosomal recessive developmental and epileptic encephalopathy, characterized by the onset of refractory seizures in infants, along with severe axial hypotonia and profoundly impaired psychomotor development. It has also been expanded to include metabolism and endocrine systems. Despite its function as a tumor suppressor gene, genetic alterations in *WWOX* have been found in several metabolic disorders and neural diseases related to brain development. Whole-exome sequencing (WES) was performed on the patient sample. Genomic DNA was fragmented, and the exons of known genes in the human genome, as well as the corresponding exon-intron boundaries,were enriched using Roche KAPA capture technology (KAPA hyperExome Library, WES identifying the homozygous variant c.406A>G in WWOX (OMIM:605131). This variant of WWOX was also observed in the prenatal WES data, indicating that both parents were heterozygous carriers and the detected variant was homozygous. This study highlighted the importance of the human *WWOX* gene in brain development and the association between *WWOX* gene mutations and developmental delay. We recommend performing WES as a primary screening before the final diagnosis, particularly in populations with high rates of consanguinity and in clinically challenging cases.

## Introduction

The WW domain-containing oxidoreductase (*WWOX*) gene is a transcriptional regulator that is expressed in the common fragile site FRA16D (16q23.1-q23.2). It encodes for 414 amino acid protein, with two interacting WW domains at the N-terminal with conserved proline and tryptophan residues and a short-chain dehydrogenase/reductase domain at the C-terminus [1-3]. The phosphorylation of Tyr33 (by tyrosine kinase, Src) activates WWOX, which then functions as a tumor suppressor gene [1,2]. WWOX performs a range of functions at the cellular, organ, and systemic levels, including metabolism, endocrine system control, and CNS differentiation and functioning. Genetic alterations in WWOX have been found in several metabolic and neural diseases. The lack of functional WWOX protein due to germline mutations impacts brain development. The severity of the disease can be determined based on complete or partial loss of protein functionality [1]. Nevertheless, most tumors show a high rate of loss of heterozygosity upon expression of somatic WWOX alternations [1]. Reduced maturation of oligodendrocytes and myelinated axons and impaired axonal conductivity have been observed in WWOX mutant mice [4]. WWOX-related syndromes have been diagnosed using whole-exome sequencing (WES) to identify the homozygous variant in WWOX (OMIM:605131), which leads to amino acid substitution. Out of 21 bioinformatics in silico experiments, 15 suggested a pathogenic effect of this variant. Additionally, based on in silico predictions, the position of the identified variant led to significant alterations in mRNA splicing owing to an altered splice site. Parallel analysis of prenatal WES data revealed that both parents were heterozygous carriers of the WWOX variant. This confirmed the homozygosity of the detected variant in the index. To the best of our knowledge, this variant has not been reported to date. Classifications of variants were conducted based on ACMG Guidelines (Richards et al.) considering database entries (inc. HGMD), Bioinformatics predictions tools, and literature status. The variant has been detected in 0.0042% of the general population (five heterozygous, 0 homozygous; gnomAD v2,1,1 controis) and this is the first time we detected it in our internal database in a homozygous state.

## Case presentation

The patient is a 21-month-old boy. He was a full-term baby delivered by cesarean section (due to a previous CS) with no history of NICU admission. The mother visited the clinic complaining that her 1-year-old son could not walk. The initial concern began when the mother brought the baby to ER after noticing an objective fever, and decreased activity and oral intake after 33 days of birth. The patient was admitted for six days and was diagnosed with an influenza A infection. He had chronic constipation, intermittently for 1 month on and off by the age of seven months. Even when the baby was one years old, he could not roll from the supine to the prone position or walk. In addition, he could not initiate a sitting or standing position. At 14 months, a mild improvement was observed according to the mother, as the baby could walk one step while holding furniture and he could roll but the concern related to his walking ability persisted. By the age of 15 months, the baby could crawl, pull to stand, sit without support, and provide good head support, but could not walk alone. Currently, at the age of 21 months, he can say 10 words but not complete sentences, climb stairs with assistance, walk, and run with recurrent falling. Upon investigating the family history, his parents were consanguineous; of his first-degree relative with four siblings, three were healthy and one was known to have seizures. His cousin also had a history of cerebral palsy.

Upon physical examination, he was vitally stable with no dysmorphic features or distress, but he had an abnormal gaze around three times, and there was a hyper-pigmented spot on his right thigh. He had good eye contact, and responded to commands and sounds but looked at non-specific objects from different angles. The power for both upper and lower limbs was 4/5 with good reflexes and tone. Bowing of the legs and flat feet were noticed.

Metabolic investigations revealed normal levels of urine organic acids and all plasma amino acids except for slightly elevated alpha aminobutyric acid and a marked decrease in cystine level (Table 1). Liver profiles, including ALT and GTT, were normal, whereas AST, albumin, total bilirubin, and direct bilirubin levels were high, and globulin level was low. The lipid profile revealed low cholesterol triglyceride levels. Anti-smooth muscle antibody test showed the following results, smooth muscle (<1.20, CMV-IgG <5, CMV IgM negative, HBsAg negative, ANA-IFA negative, and antiHBs 699.89). The TSH and free T4 levels were within the normal range. Creatine kinase was high. 25-OH vitamin D and vitamin B12 levels were high. Abdominal ultrasonography revealed mild hepatosplenomegaly. Liver approximately 6.6 cm, with no focal lesion, and no intra-or extrahepatic biliary duct dilatation was observed. His hepatic and portal veins were patent. The gallbladder was partially contracted and appeared on gross examination. There was no abdominopelvic lymphadenopathy. Brain MRI results were normal.

**Table 1:** Investigations value

Investigations value	Value
Alpha aminobutyric	High
Cystine	Low
AST	High
ALT	Low
GGT	Low
Albumin	High
Total Bilirubin	High
Direct Bilirubin	High
Globulin	Low
Triglyceride	Low
Creatinine Kinase	High
Vitamin B12	High
25-OH vitamin D	High

The patient samples' peripheral blood was sent for WES analyzed at Bioscientia Labor Ingelheim. Genomic DNA was fragmented, and the exons and their exon-intron boundaries of the known genes in the human genome were enriched using Roche KAPA capture technology (KAPA HyperExome Library), amplified, and sequenced simultaneously by Illumina technology (next-generation sequencing, NGS). The target regions were sequenced, with an average coverage of 116.4-fold. Approximately 99.9% of the regions of interest had 15-fold coverage; for approximately 99.9%, a 20-fold coverage was obtained. WES identified the homozygous variant c.406A>G in WWOX (OMIM:605131) resulting in amino acid change (Table 2). Out of 21 bioinformatic in silico experiments, 15 showed a pathogenic effect of this variant. The in silico predicted that the position of the identified variant might lead to significant alterations in mRNA splicing owing to an altered splice site. A parallel analysis of prenatal WES data revealed that both parents were heterozygous carriers of the *WWOX *variant, confirming its homozygosity.

**Table 2:** Amino acid analysis

Metabolism diagnostics	Result	Unit	Reference range
Phosphoserine	11		1-20
Taurine	54		15-143
Phosphoethanolamine	5		<6
Aspartic Acid	7		<23
Hydroxyproline	17		<63
Threonine	137		24-174
Serine	144		71-186
Asparagine	60		21-95
Glutamic Acid	56		10-133
Glutamine	423		246-1182
Sarcosine	0		Not detected
Alpha-Aminoadipic Acid	0		Not detected
Proline	147		52-298
Glycine	166		81-436
Alanine	276		143-439
Citrulline	26		3-53
Alpha Aminobutyric Acid	28*		3-26
Valine	254		64-294
Cystine	5*		16-84
Methionine	25		9-42
Cystathionine	0		<5
Isoleucine	78		31-86
Leucine	130		47-155
Tyrosine	67		22-108
Phenylalanine	56		31-75
Beta Alanine	0		<7
Beta Aminoisobutyric Acid	0		Not detected
Gamma Aminobutyric Acid	0		Not detected
Ethanolamine	0		<4
Tryptophan	48		23-71
Hydroxylysine	0		<7
Ornithine	75		22-103
Lysine	181		52-196

## Discussion

*WWOX* gene alterations can cause severe problems. It is expressed in the common fragile site FRA16D (16q23.1 - q23.2), prone to high frequencies of loss of heterozygosity and homozygous deletion [5]. WWOX expression has been uniformly detected in neurons and glial cell types. Moreover, progenitor oligodendrocytes are considered to have higher WWOX expression compared to a mature myelinated oligodendrocyte. WWOX is involved in the development and differentiation of these specialized cells. Stable expression of WWOX in microglial cells has been observed from the analysis of RNA-seq data available in the public database [6-8]. Severe neural diseases, metabolic disorders, and early death can result from a homozygous null mutation in the *WWOX* gene [9]. The present case report study reported the first case of a WWOX-related phenotype. This WWOX variant is found in 0.0042% of the general population (5 heterozygous, 0 homozygous; gnomAD v2,1,1 controis); to the best of our knowledge, first time detected in a homozygous state. It has been reported that 87% of the variants in gnomAD appeared 1-109 times, with an average of 14 individual alleles per variant. None of these variants were observed in a homozygous state [3]. Pathogenic variants in WWOX cause autosomal recessive developmental and epileptic encephalopathy 28 (DEE28; OMIM:6162111), characterized by the onset of refractory seizures in infants. Affected individuals have severe axial hypotonia and severely impaired psychomotor development. More severely affected patients have acquired microcephaly, poor or absent visual contact, and retinal degeneration leading to early death. From the available information, the patient phenotype appears partially supportive of DEE28. In the present study, our patient presented with a global developmental delay and no early seizure disorder despite a family history of seizures and cerebral palsy in his brother and cousin, respectively. Additionally, the MRI was normal, with no progressive microcephaly or bilateral optic atrophy. In addition, our patient had mild hepatosplenomegaly and a high abnormal level of creatinine kinase (CK), cholestasis, and chronic constipation that have not been reported in WWOX patients; nevertheless, the symptoms in our patient are considered less severe than the other variants that have been reported previously. A case in the Emirates, for instance, described an infant with an early seizure disorder associated with global developmental delay, progressive microcephaly, delayed psychomotor development, and spastic quadriplegia [10]. This phenotype is very similar to another case reported in an Egyptian family; however, it is considered a more lethal case as the patient died at the age of 16 months [11]. The patients in the other two reported families (Saudi and Palestinian) did not develop progressive microcephaly, and the severity of the disease was considerably less than that in the Emiratis and Egyptian cases. Furthermore, the case of the Saudi Family lacked spasticity and the deep tendon reflexes were diminished, whereas the case of the Palestinian had spasticity with exaggerated reflexes in addition to the ataxia (Table 3) [12].

**Table 3:** Details regarding the cases that have been compared

Study	Current case, 2021	Ehaideb et al. [2]	Abdel-Salam et al. [11]	Mallaret et al. [12]	Ben-Salem et al. [10]
Number of patients	1	3	1	3	1
Gender	Male	Male	Female		Male
		Male			
		Female			
Nucleotide change	c.406A>G	c.409+1G > T	c.160G > T	c.1114G>C	
		c.160G > T			
		c.160G > T			
Variant	Chr16:78149048	Ch16:78149052 G>T	chr16:25,457,305-79,894,309; GRCh37		
Amino acid change	P.lle136Val	-	p.Arg54	p.Gly372Arg	
		p.Arg54			
		p.Arg54			
Nature of mutation	Homozygous	Homozygous	Homozygous	Homozygous	Homozygous
		Homozygous			
		Homozygous			
Ethnicity	Saudi	Saudi	Egyptian	Palestinian	Emirati
Age	21 months	1 year & 9 months		2 years	5 months
		3 Months			
		18 months			
Brain MRI	Normal	Brain Atrophy, increased white matter signal in cerebellar area	supratentorial atrophy, hypoplasia of the hippocampus and the temporal lobe, widened subarachnoidal space, thin hypoplastic corpus callo- sum		Cerebral atrophy, increased CSF in subarachnoid space, polymicrogyria on right frontopareital region
		brain atrophy, moderate corpus callosum thinning			
		generalized asymmetrical brain tissue volume loss and asymmetrical ventricular dilatation, thinning of corpus callosum and periventricular white matter more left side			
Clinical manifestations	Developmental delay , chronic constipation , abnormal high level creatinine kinase (CK) cholestasis and low lipied profile, hyper-pigmented ( café au lait ) spot in his right thigh.	Seizure, developmental delay, kyphosis, , scoliosis, horizontal nystagmus, decreased deep tendon reflexes and axial hypotoni	Epilepsy, optic atrophy, abnormal retinal pigmentation & mouth movement, psychomotor delay, microcephaly, growth retardation.	Generalized tonic-clonic epilepsy, mental retardation, ataxia, upper motor neuron affection, leg spasticity.	Microcephaly, seizure, spasticity, horizontal nystagmus, bilateral optic atrophy, right hearing loss, delayed psychomotor development
		Infantile Spasm, decreased activity & interaction, axial hypotonia, spastic, and peripheral hypertonia			
		Hypoactive, abnormal movements, vomiting, spastic, hypertonic, contractures of achilles tendon and not responding to voices			
Status of patient	Alive		Died at 16 months		

WWOX is critical for homeostasis in vivo and cell death. The point or homozygous nonsense mutations in the *WWOX *gene affect embryonic neural development, resulting in autosomal recessive cerebellar ataxia and epilepsy, growth retardation, microcephaly with seizure, retinal degeneration, and early death at 16 months of age [9]. As shown in our case, the lipid profile was low, which can be attributed to the *WWOX* gene, as it has been reported to be involved in the regulation of lipid homeostasis and metabolism [13,14]. Thus, WWOX plays a crucial role in neural development and lipid metabolism. The lack of functional WWOX can lead to severe neural diseases and metabolic disorders. Moreover, the severity of phenotypes depends on the genetic makeup of the patient. Unfortunately, the number of cases with WWOX mutations is low; therefore, it remains uncertain whether some *WWOX *mutations have a unique phenotype. WWOX genotypes appeared to correlate with the severity and onset of seizures and spasticity in the studied cases. However, studies on more patients are needed to establish accurate genotype-phenotype correlations. Given the function of *WWOX* as a signaling protein involved in different protein-protein interactions, the disruption of neuronal pathways could explain the susceptibility to seizures [4].

## Conclusions

We identified a consanguineous Saudi family with the homozygous variant c.406A>G in WWOX (OMIM:605131), which led to amino acid substitution. Both parents were heterozygous carriers of the *WWOX* variant. This confirmed the homozygosity of the detected variant in the index. The child presented with developmental delay, abnormally high levels of CK, cholestasis, and a low lipid profile. Our analysis showed that the loss of function of WWOX could be responsible for these reported concerns. It also highlights the role of WWOX in neurodevelopment and supports the genotypic and phenotypic variability of this condition. Therefore, it is essential to establish accurate genotype/phenotype correlations in patients. We recommend performing WES as an early diagnostic test to facilitate the final diagnosis, particularly in populations with high rates of consanguinity and in clinically challenging cases.
